# Treatment patterns and survival in T4b esophageal cancer: a retrospective cohort study

**DOI:** 10.18632/aging.205747

**Published:** 2024-04-18

**Authors:** Bin-Bin Yu, Jiang-Qiong Huang, Huan-Wei Liang, Yang Liu, Long Chen, Su Pei, Wei Huang, Xin-Bin Pan

**Affiliations:** 1 Department of Radiation Oncology, Guangxi Medical University Cancer Hospital, Nanning 530021, Guangxi, P.R. China

**Keywords:** esophageal cancer, T4b, treatment patterns, survival

## Abstract

Purpose: This study aims to evaluate the efficacy of various treatment approaches in stage T4b esophageal cancer patients.

Materials and methods: Data were extracted from the Surveillance, Epidemiology, and End Results databases, covering patients diagnosed with esophageal cancer between 2000 and 2020. Kaplan-Meier analysis was used to assess cancer-specific survival (CSS) and overall survival (OS) across different treatment patterns.

Results: The study included 482 patients: 222 (46.1%) received chemoradiotherapy, 58 (12.0%) underwent radiotherapy alone, 37 (7.7%) received chemotherapy alone, 50 (10.4%) underwent surgery, and 115 (23.8%) received no treatment. Median CSS were 12, 4, 6, 18, and 1 month for chemoradiotherapy, radiotherapy alone, chemotherapy alone, surgery, and non-treatment groups. Median OS for these groups were 11, 3, 6, 17, and 1 month, respectively. Multivariable proportional hazard regression analysis revealed that patients who underwent surgery experienced significantly improved CSS (hazard ratio [HR] = 0.42, 95% confidence interval [CI]: 0.24-0.72; P = 0.002) and OS (HR = 0.45, 95% CI: 0.28-0.74; P = 0.002) compared to those receiving chemoradiotherapy after propensity score matching.

Conclusions: Esophagectomy, with or without radiotherapy and/or chemotherapy, results in better survival outcomes than chemoradiotherapy in patients with stage T4b esophageal cancer.

## INTRODUCTION

Esophageal cancer is the sixth leading cause of cancer-related mortality worldwide [1]. This malignancy poses a substantial challenge, primarily due to ineffective methods for early detection and screening. Consequently, most patients receive their diagnosis at an advanced stage [2]. For those with resectable disease, the established treatment protocol involves neoadjuvant chemoradiotherapy followed by esophagectomy [3–8]. In cases of unresectable disease, the preferred treatment is concurrent chemoradiotherapy [9, 10].

Particularly in locally advanced cases, stage T4b esophageal cancer represents a dire situation, characterized by tumor invasion into critical structures such as the aorta, vertebral body, and trachea. The prognosis for patients at this stage has historically been poor, with a median overall survival (OS) of less than 10 months, mainly due to limited effective treatment options [11–15].

Managing stage T4b esophageal cancer is highly challenging. Treatment choices are restricted, and these cases are often excluded from most clinical trials [8, 16–19]. This exclusion has led to considerable debate over the best treatment approach for patients at this stage. Our study aims to illuminate the treatment strategies and associated clinical outcomes in stage T4b esophageal cancer patients, addressing a crucial gap in current medical understanding.

## RESULTS

### Patient characteristics

**Figure 1:**
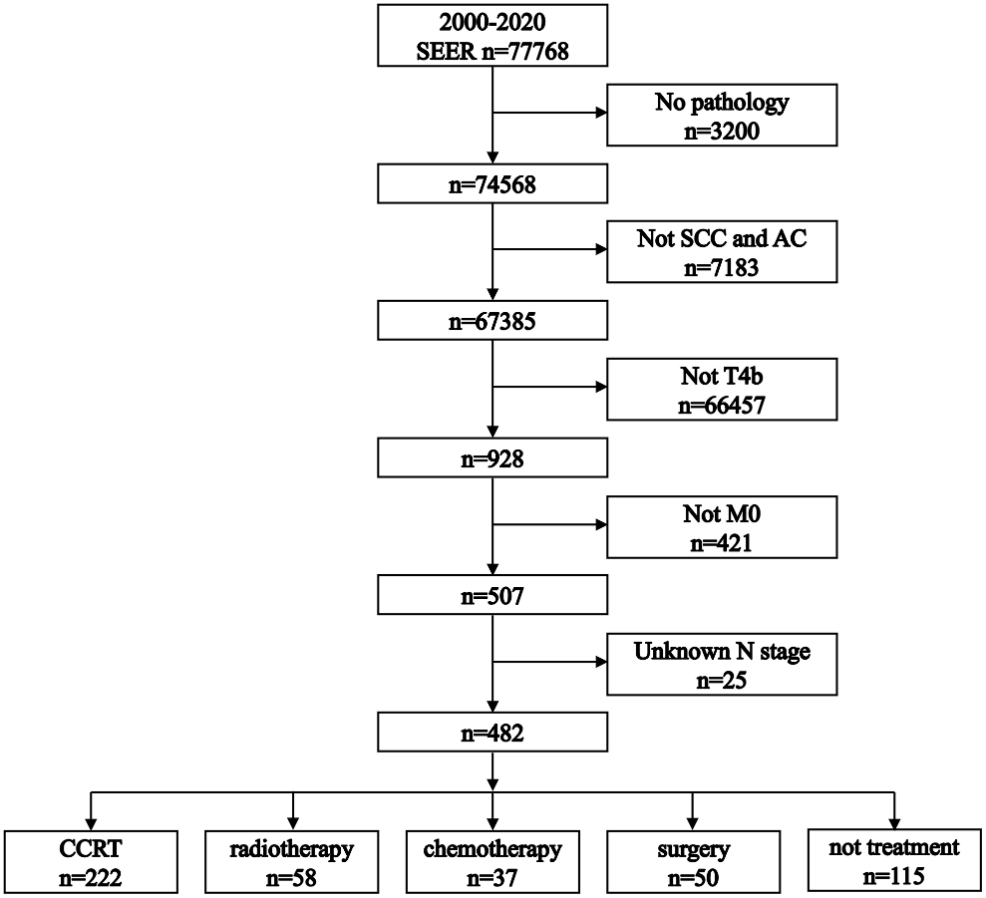
**Flowchart illustrating the process of patient selection.** SCC: squamous cell carcinoma. AC: adenocarcinoma.

As illustrated in Figure 1, our initial investigation involved 77,768 patients with esophageal cancer. After applying the inclusion criteria, 482 patients were eligible for the study. Among these, 222 (46.1%) received chemoradiotherapy, 58 (12.0%) underwent radiotherapy alone, 37 (7.7%) were treated with chemotherapy alone, 50 (10.4%) underwent surgery, and 115 (23.8%) received no treatment.

Table 1 presents a summary of the patient characteristics. Baseline characteristics like sex, tumor grade, and N stage were comparably balanced across the treatment groups. However, differences were observed in age, race, primary site, and histological types among the groups.

**Table 1:** Patient characteristics.

	**Chemoradiotherapy**	**Radiotherapy**	**Chemotherapy**	**Surgery**	**Not treatment**	**P**
	**(n=222)**	**(n=58)**	**(n=37)**	**(n=50)**	**(n=115)**	
Age						0.031
<65	121 (54.5%)	21 (36.2%)	21 (56.8%)	26 (52.0%)	47 (40.9%)	
≥65	101 (45.5%)	37 (63.8%)	16 (43.2%)	24 (48.0%)	68 (59.1%)	
Sex						0.503
female	66 (29.7%)	18 (31.0%)	9 (24.3%)	14 (28.0%)	43 (37.4%)	
male	156 (70.3%)	40 (69.0%)	28 (75.7%)	36 (72.0%)	72 (62.6%)	
Race						0.001
white	147 (66.2%)	26 (44.8%)	24 (64.9%)	40 (80.0%)	70 (60.9%)	
black	54 (24.3%)	28 (48.3%)	9 (24.3%)	2 (4.0%)	33 (28.7%)	
others	21 (9.5%)	4 (6.9%)	4 (10.8%)	8 (16.0%)	12 (10.4%)	
Site						0.025
upper third	61 (27.5%)	15 (25.9%)	7 (18.9%)	9 (18.0%)	33 (28.7%)	
Middle third	73 (32.8%)	25 (43.1%)	12 (32.4%)	21 (42.0%)	31 (27.0%)	
lower third	57 (25.7%)	8 (13.8%)	10 (27.1%)	15 (30.0%)	19 (16.5%)	
overlapping	31 (14.0%)	10 (17.2%)	8 (21.6%)	5 (10.0%)	32 (27.8%)	
Histology						0.001
SCC	179 (80.6%)	51 (87.9%)	25 (67.6%)	32 (64.0%)	101 (87.8%)	
AC	43 (19.4%)	7 (12.1%)	12 (32.4%)	18 (36.0%)	14 (12.2%)	
Grade						0.192
I/II	108 (48.6%)	22 (38.0%)	15 (40.6%)	22 (44.0%)	46 (40.0%)	
III/IV	74 (33.4%)	18 (31.0%)	10 (27.0%)	21 (42.0%)	42 (36.5%)	
unknown	40 (18.0%)	18 (31.0%)	12 (32.4%)	7 (14.0%)	27 (23.5%)	
N stage						0.540
N0	76 (34.2%)	21 (36.2%)	13 (35.1%)	13 (26.0%)	47 (40.9%)	
N1	110 (49.5%)	27 (46.6%)	18 (48.7%)	25 (50.0%)	56 (48.7%)	
N2	24 (10.8%)	9 (15.5%)	3 (8.1%)	9 (18.0%)	7 (6.0%)	
N3	12 (5.5%)	1 (1.7%)	3 (8.1%)	3 (6.0%)	5 (4.4%)	

SCC, squamous cell carcinoma; AC, adenocarcinoma.

The median follow-up times varied: 11 months (interquartile range [IQR]: 5-21 months) for the chemoradiotherapy group, 3 months (IQR: 2-6 months) for the radiotherapy alone group, 6 months (IQR: 4-11 months) for the chemotherapy alone group, 17 months (IQR: 8-38 months) for the surgery group, and 1 month (IQR: 0-2 months) for the non-treatment group.

### Cancer-specific survival

**Figure 2:**
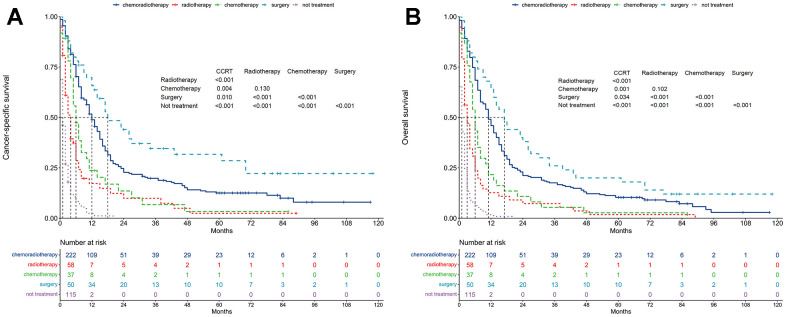
**Survival between treatment patterns.** (**A**) Cancer-specific survival. (**B**) Overall survival.

The median CSS differed across groups: chemoradiotherapy (12 months), radiotherapy alone (4 months), chemotherapy alone (6 months), surgery (18 months), and non-treatment (1 month) (Figure 2A). The 1-year CSS rates were 48.8% (chemoradiotherapy), 17.3% (radiotherapy alone), 23.7% (chemotherapy alone), 65.7% (surgery), and 2.4% (non-treatment). CSS showed significant differences in pairwise comparisons, except between radiotherapy alone and chemotherapy alone groups (P = 0.130).

Unadjusted analysis, with chemoradiotherapy as the reference, revealed that radiotherapy alone (HR = 2.28, 95% CI: 1.66-3.14; P < 0.001), chemotherapy alone (HR = 1.65, 95% CI: 1.14-2.39; P = 0.008), and non-treatment (HR = 7.10, 95% CI: 5.42-9.31; P < 0.001) were adverse prognostic factors for CSS (Table 2).

**Table 2:** Univariable proportional hazards regressions.

	**Cancer-specific survival**			**Overall survival**		
	**HR**	**95% CI**	**P**	**HR**	**95% CI**	**P**
Age						
<65	reference			reference		
≥65	1.00	0.82-1.22	0.996	1.01	0.84-1.21	0.953
Sex						
female	reference			reference		
male	1.11	0.90-1.38	0.325	1.12	0.91-1.37	0.280
Race						
white	reference			reference		
black	1.35	1.08-1.69	0.009	1.39	1.12-1.71	0.003
others	0.97	0.70-1.34	0.843	0.89	0.65-1.23	0.491
Site						
upper third	reference			reference		
middle third	0.89	0.69-1.15	0.371	0.95	0.75-1.21	0.669
lower third	0.82	0.62-1.08	0.161	0.81	0.62-1.05	0.114
overlapping	1.26	0.94-1.70	0.118	1.30	0.98-1.72	0.066
Histology						
SCC	reference			reference		
AC	0.76	0.60-0.97	0.027	0.74	0.58-0.93	0.009
Grade						
I/II	reference			reference		
III/IV	1.16	0.93-1.44	0.192	1.17	0.95-1.44	0.135
unknown	1.12	0.87-1.45	0.372	1.12	0.88-1.43	0.373
N stage						
N0	reference			reference		
N1	0.95	0.76-1.18	0.622	0.86	0.70-1.05	0.142
N2	1.07	0.76-1.50	0.710	1.05	0.77-1.45	0.749
N3	1.08	0.69-1.69	0.750	1.01	0.66-1.56	0.955
Treatment						
chemoradiotherapy	reference			reference		
radiotherapy	2.28	1.66-3.14	<0.001	2.42	1.79-3.26	<0.001
chemotherapy	1.65	1.14-2.39	0.008	1.72	1.21-2.45	0.002
surgery	0.64	0.45-0.92	0.015	0.72	0.52-0.99	0.047
not treatment	7.10	5.42-9.31	<0.001	7.32	5.64-9.49	<0.001

SCC, squamous cell carcinoma; AC, adenocarcinoma; HR, hazard ratio; CI, confidence interval.

Conversely, surgery was a positive prognostic factor for CSS (HR = 0.64, 95% CI: 0.45-0.92; P = 0.015).

**Figure 3:**
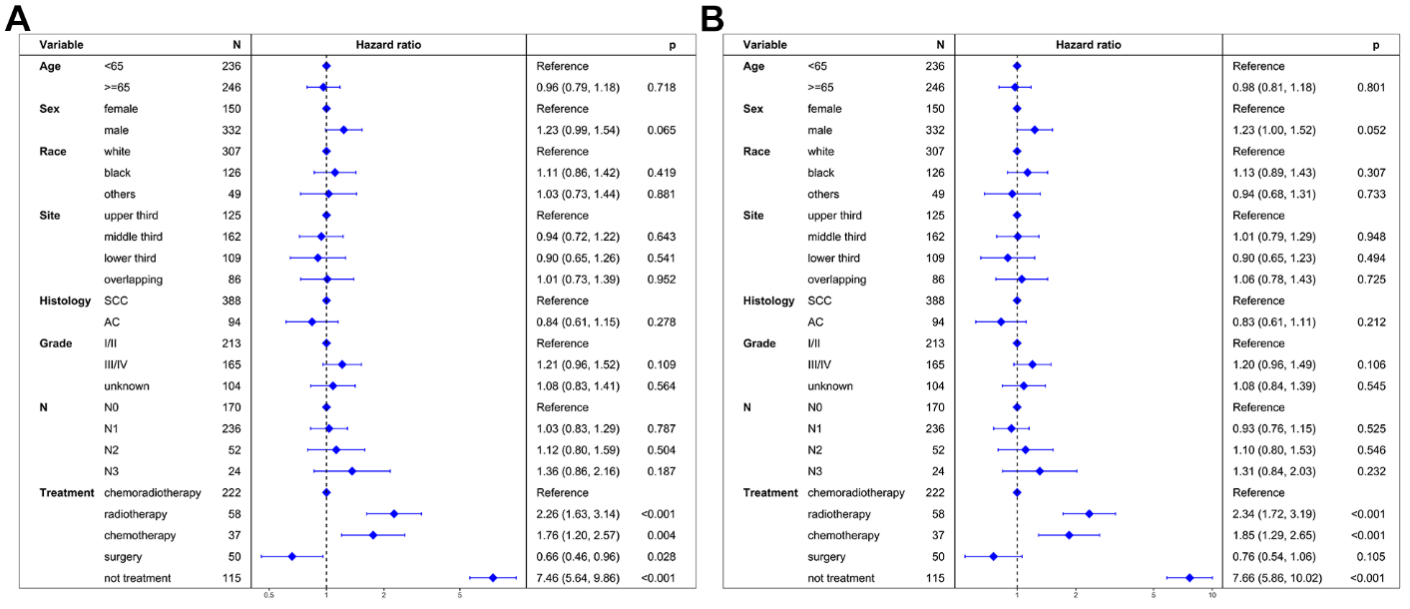
**Multivariate regression analysis of prognostic factors.** (**A**) Cancer-specific survival. (**B**) Overall survival. SCC: squamous cell carcinoma. AC: adenocarcinoma.

Multivariable proportional hazard regression analysis confirmed that radiotherapy alone (HR = 2.26, 95% CI: 1.63-3.14; P < 0.001), chemotherapy alone (HR = 1.76, 95% CI: 1.20-2.57; P = 0.004), and non-treatment (HR = 7.46, 95% CI: 5.64-9.86; P < 0.001) were independent adverse prognostic factors for CSS (Figure 3A). In contrast, surgery was an independent positive prognostic factor for CSS (HR = 0.66, 95% CI: 0.46-0.96; P = 0.028).

### Overall survival

The median OS differed across groups: chemoradiotherapy (11 months), radiotherapy alone (3 months), chemotherapy alone (6 months), surgery (17 months), and non-treatment (1 month) (Figure 2B). The 1-year OS rates were 46.0% (chemoradiotherapy), 12.7% (radiotherapy alone), 21.6% (chemotherapy alone), 64.0% (surgery), and 1.8% (non-treatment). OS differed significantly among the groups, except between radiotherapy alone and chemotherapy alone groups (P = 0.102).

Unadjusted analysis, with chemoradiotherapy as the reference, revealed that radiotherapy alone (HR = 2.42, 95% CI: 1.79-3.26; P < 0.001), chemotherapy alone (HR = 1.72, 95% CI: 1.21-2.45; P = 0.002), and non-treatment (HR = 7.32, 95% CI: 5.64-9.49; P < 0.001) were adverse prognostic factors for OS (Table 2). Conversely, surgery was a positive prognostic factor for OS (HR = 0.72, 95% CI: 0.52-0.99; P = 0.047).

Multivariable proportional hazard regression analysis further confirmed that radiotherapy alone (HR = 2.34, 95% CI: 1.72-3.19; P < 0.001), chemotherapy alone (HR = 1.85, 95% CI: 1.29-2.65; P < 0.001), and non-treatment (HR = 7.66, 95% CI: 5.86-10.02; P < 0.001) were independent adverse prognostic factors for OS (Figure 3B). However, surgery was not an independent prognostic factor for OS (HR = 0.76, 95% CI: 0.54-1.06; P = 0.105).

### Cancer-specific survival between chemoradiotherapy and surgery groups after PSM

Table 3 summarizes the patient characteristics between the chemoradiotherapy and surgery groups after PSM. Patient characteristics were well balanced across all covariates after PSM (P > 0.05).

**Table 3:** Patient characteristics between chemoradiotherapy and surgery groups after propensity score matching.

	**Chemoradiotherapy (n=46)**	**Surgery (n=46)**	**P**
Age			0.675
<65	27 (58.7%)	24 (52.2%)	
≥65	19 (41.3%)	22 (47.8%)	
Sex			0.821
female	15 (32.6%)	13 (28.3%)	
male	31 (67.4%)	33 (71.7%)	
Race			0.066
white	41 (89.1%)	37 (80.4%)	
black	4 (8.7%)	2 (4.4%)	
others	1 (2.2%)	7 (15.2%)	
Site			0.958
upper third	7 (15.2%)	9 (19.6%)	
middle third	20 (43.5%)	19 (41.3%)	
lower third	14 (30.4%)	14 (30.4%)	
overlapping	5 (10.9%)	4 (8.7%)	
Histology			1.000
SCC	31 (67.4%)	31 (67.4%)	
AC	15 (32.6%)	15 (32.6%)	
Grade			0.682
I/II	24 (52.2%)	21 (45.7%)	
III/IV	14 (30.4%)	18 (39.1%)	
unknown	8 (17.4%)	7 (15.2%)	
N stage			0.803
N0	12 (26.1%)	13 (28.3%)	
N1	24 (52.2%)	25 (54.3%)	
N2	9 (19.5%)	6 (13.0%)	
N3	1 (2.2%)	2 (4.4%)	

SCC, squamous cell carcinoma; AC, adenocarcinoma.

**Figure 4:**
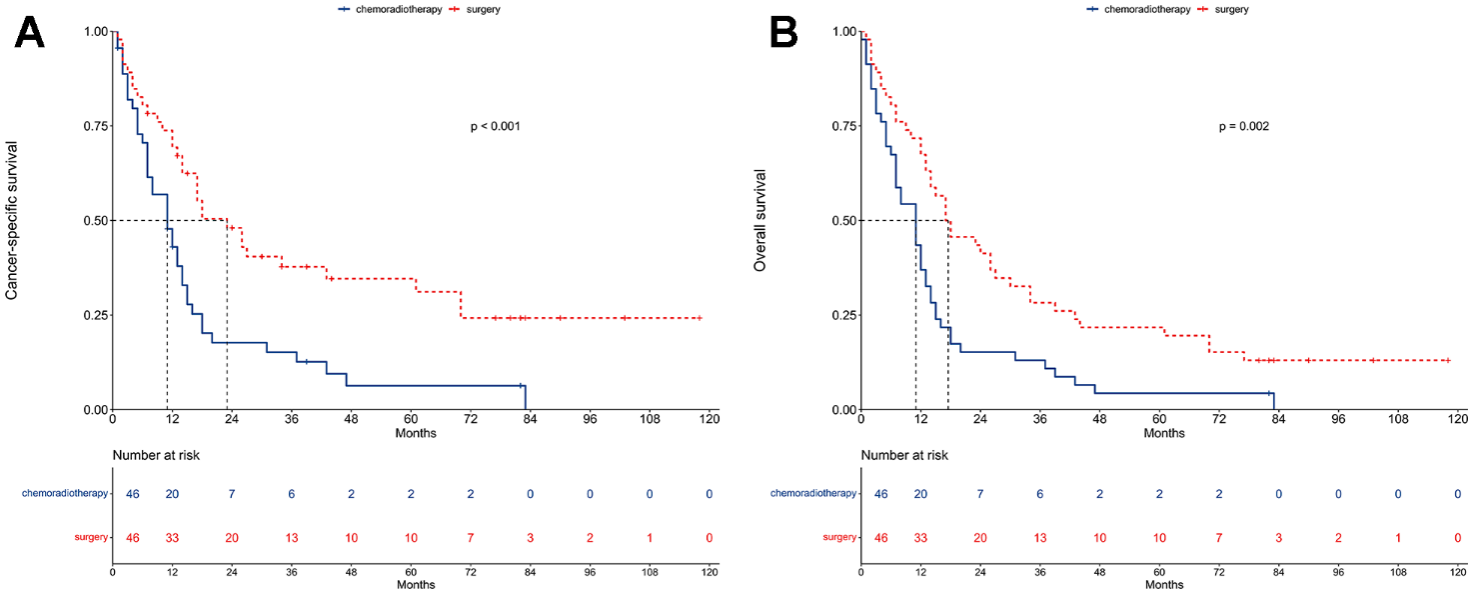
**Survival between chemoradiotherapy and surgery groups after propensity score matching.** (**A**) Cancer-specific survival. (**B**) Overall survival.

The median CSS was 11 and 23 months for the chemoradiotherapy and surgery groups (Figure 4A). The 3-year CSS was 15.2% and 37.8% for the chemoradiotherapy and surgery groups. The 5-year CSS was 6.3% and 34.6% for the chemoradiotherapy and surgery groups.

**Figure 5:**
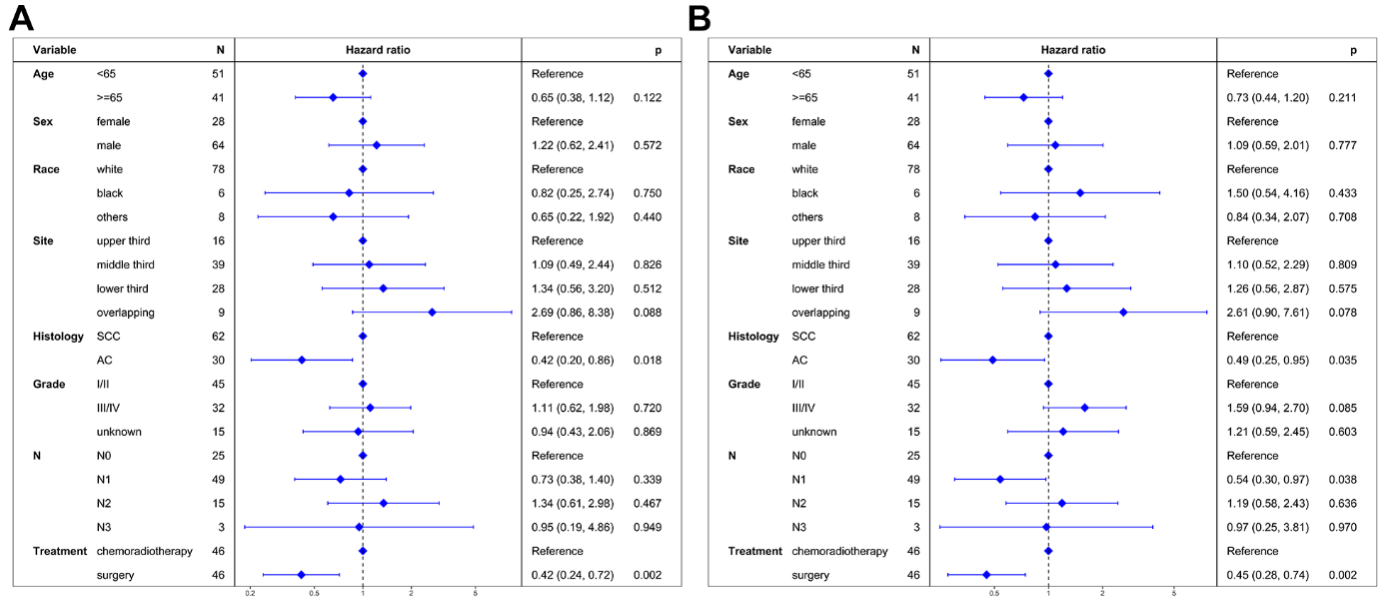
**Multivariate regression analysis of prognostic factors after propensity score matching.** (**A**) Cancer-specific survival. (**B**) Overall survival. SCC: squamous cell carcinoma. AC: adenocarcinoma.

Univariable proportional hazards regressions revealed that patients undergoing surgery had better CSS compared to those receiving chemoradiotherapy (HR = 0.45, 95% CI: 0.28-0.73; P < 0.001). Multivariable proportional hazard regression analysis further confirmed that surgery was an independent prognostic factor for CSS (HR = 0.42, 95% CI: 0.24-0.72; P = 0.002; Figure 5A).

### Overall survival between chemoradiotherapy and surgery groups after PSM

The median OS was 11 and 17.5 months for the chemoradiotherapy and surgery groups (Figure 4B). The 3-year OS was 13.0% and 28.3% for the chemoradiotherapy and surgery groups. The 5-year OS was 4.4% and 21.7% for the chemoradiotherapy and surgery groups.

Univariable proportional hazards regressions revealed that patients undergoing surgery had better OS compared to those receiving chemoradiotherapy (HR = 0.51, 95% CI: 0.33-0.78; P = 0.002). Multivariable proportional hazard regression analysis further confirmed that surgery was an independent prognostic factor for OS (HR = 0.45, 95% CI: 0.28-0.74; P = 0.002; Figure 5B).

## DISCUSSION

Stage T4b esophageal cancer represents a uniquely challenging subgroup within locally advanced diseases, characterized by tumor invasion into adjacent major structures, which typically precludes esophagectomy as a treatment option [9, 10]. However, our study suggests that carefully selected patients with stage T4b esophageal cancer who undergo surgery with or without radiotherapy and/or chemotherapy exhibit improved CSS and OS compared to those receiving chemoradiotherapy. These findings indicate that well-selected stage T4b esophageal cancer patients may be candidates for esophagectomy, potentially extending their survival.

Definitive chemoradiotherapy remains the standard treatment for inoperable stage T4b esophageal cancer [3–8]. However, recent research has explored the use of induction therapies, including chemoradiotherapy or chemotherapy, with the aim of making surgery a viable option for these patients [15, 20]. Notably, patients who underwent esophagectomy, in combination with radiotherapy and/or chemotherapy, showed a remarkable median OS of 43.9 months [11]. This outcome was markedly better than the median OS of 11.4 months observed in patients who received definitive chemoradiotherapy [12].

A key factor in the observed superior OS among patients who underwent esophagectomy, compared to those receiving definitive chemoradiotherapy, appears to be patient selection. Notably, the group receiving definitive chemoradiotherapy often had higher comorbidities and poorer performance status, potentially leading to poorer survival outcomes. Additionally, the development of esophageal fistulas, which occurred in 30.1% of stage T4b patients undergoing definitive chemoradiotherapy [21], might also contribute to the improved OS observed in the surgery group. These aspects underscore the importance of considering a comprehensive range of factors, including treatment efficacy, safety, and potential complications, in determining the most appropriate treatment strategy.

Indeed, our study reported a median OS of 11 months for patients receiving chemoradiotherapy, consistent with previous findings [12]. However, the median OS of 17 months observed in the surgery group of our study was significantly lower than the 43.9 months reported by Cushman et al. [11]. This discrepancy in OS can be attributed to differences in the treatment approaches between the two studies. Cushman et al. [11] exclusively included patients who received neoadjuvant chemotherapy or chemoradiotherapy, which is known to be associated with improved outcomes in esophageal cancer patients. In contrast, our study included a subset of patients who underwent adjuvant therapies post-esophagectomy without preceding neoadjuvant chemoradiotherapy or chemotherapy. Given the relatively small size of our surgery group, it was impractical to accurately calculate the median OS for patients who received neoadjuvant therapies, due to the limited statistical power available for such analysis.

While our study demonstrates improved CSS and OS among the surgery group for stage T4b esophageal cancer, caution is necessary when considering surgery for all cases in this stage. Stage T4b esophageal cancer is a heterogeneous group. Success of esophagectomy heavily depends on careful patient selection. Surgery is not feasible for instances involving invasion into the aorta or airway. Consequently, the surgical subgroup often represents less invasive cases. Additionally, survival in stage T4b is significantly influenced by comorbidities and performance status, which in turn affect treatment tolerance. Patients with better performance status and fewer comorbidities are more likely candidates for surgery, potentially leading to more favorable outcomes. However, the limitations of the SEER database preclude detailed analysis of these factors, warranting further research to assess their prognostic impact.

To identify patients who might benefit from esophagectomy, several criteria are useful. First, those showing complete responses or partial responses to neoadjuvant chemoradiotherapy are prime candidates for surgery [7, 8]. Second, patients with tumors deemed technically resectable by surgeons are also suitable [22]. Third, a clinical stage of N0 is preferable [23, 24] as the primary treatment failure in these patients is local recurrence, and primary tumor resection can offer better local control. However, it would be premature to claim that surgery is the optimal choice for all suitable cases without further evidence from large-scale clinical trials.

While this study demonstrated the potential benefits of esophagectomy for stage T4b esophageal cancer patients, it was limited by the relatively small sample size of the surgery group, which comprised 10.4% of the total patient cohort. This small sample size reduces the statistical power of the analysis. We employed various analytical methods, including multivariate adjustment and PSM, to mitigate potential biases. Consistently, both pre-PSM and post-PSM multivariate analyses indicated superior CSS and OS in the surgery group compared to chemoradiotherapy. However, larger-scale studies across diverse healthcare centers are necessary for further validation.

In conclusion, this study highlights that surgery with or without radiotherapy and/or chemotherapy can improve survival outcomes in stage T4b esophageal cancer patients, compared to chemotherapy or radiotherapy alone. Careful patient selection for esophagectomy is key, potentially offering enhanced treatment outcomes over chemoradiotherapy alone.

## MATERIALS AND METHODS

### Patients

The study included patients diagnosed with esophageal cancer in the Surveillance, Epidemiology, and End Results (SEER) databases from 2000 to 2020, meeting the following criteria: (1) aged 18 years or older, (2) histopathological confirmation of adenocarcinoma (SEER codes: 8140-8389, according to the International Classification of Diseases for Oncology, 3rd Edition) or squamous cell carcinoma (SEER codes: 8050-8089), (3) T4b classification, (4) N0-3 stage, and (5) M0 stage. Extracted patient characteristics included age, gender, race, primary site, histological type, tumor grade, N stage, and treatment patterns.

### Treatment patterns

Based on the SEER database, patients with T4bN0-3M0 stage esophageal cancer were categorized into five treatment groups: chemoradiotherapy, radiotherapy alone, chemotherapy alone, surgery, or non-treatment. The chemoradiotherapy group received local-regional radiotherapy combined with chemotherapy. The radiotherapy alone group underwent only local-regional radiotherapy. Patients in the chemotherapy alone group were given chemotherapy exclusively. The surgery group underwent surgery with or without radiotherapy and/or chemotherapy. The non-treatment group received no anti-cancer treatments.

### Statistical analysis

Age was categorized based on median values. Categorical variables, such as age, gender, race, primary site, histological types, tumor grade, and N stage, were compared across different treatment patterns using the χ2 test or Fisher’s exact test.

Cancer-specific survival (CSS) and OS were analyzed using Kaplan-Meier methods, with log-rank tests for treatment pattern comparisons. Pairwise comparisons were made among treatment groups. Univariable proportional hazards regression analysis identified potential prognostic factors. Multivariable proportional hazards regressions, adjusting for age, gender, race, primary site, histological types, tumor grade, N stage, and treatment patterns, determined independent prognostic factors. Results were expressed as hazard ratios (HRs) with 95% confidence intervals (CIs).

To minimize selection bias between chemoradiotherapy and surgery groups, a matched case-control analysis using propensity score matching (PSM) was conducted. The surgery decision was the dependent variable for calculating the propensity score. One-to-one matching without replacement was performed using nearest-neighbor matching on the logit of the propensity score, considering confounding factors like age, gender, race, primary site, histological types, tumor grade, and N stage. A caliper width of 0.02 was used.

Statistical analyses were performed using SPSS Statistics Version 26.0 (IBM Co., Armonk, NY, USA) and R software (version 4.2.2). Statistical significance was established with two-tailed P-values less than 0.05.

### Availability of data and material

The data are available from the corresponding author upon request.
